# Development of medical device software for the screening and assessment of depression severity using data collected from a wristband-type wearable device: SWIFT study protocol

**DOI:** 10.3389/fpsyt.2022.1025517

**Published:** 2022-12-21

**Authors:** Taishiro Kishimoto, Shotaro Kinoshita, Toshiaki Kikuchi, Shogyoku Bun, Momoko Kitazawa, Toshiro Horigome, Yuki Tazawa, Akihiro Takamiya, Jinichi Hirano, Masaru Mimura, Kuo-ching Liang, Norihiro Koga, Yasushi Ochiai, Hiromi Ito, Yumiko Miyamae, Yuiko Tsujimoto, Kei Sakuma, Hisashi Kida, Gentaro Miura, Yuko Kawade, Akiko Goto, Fumihiro Yoshino

**Affiliations:** ^1^Hills Joint Research Laboratory for Future Preventive Medicine and Wellness, Keio University School of Medicine, Tokyo, Japan; ^2^i2medical LLC, Kawasaki, Japan; ^3^Graduate School of Interdisciplinary Information Studies, The University of Tokyo, Tokyo, Japan; ^4^Department of Neuropsychiatry, Keio University School of Medicine, Tokyo, Japan; ^5^Sato Hospital, Yamagata, Japan; ^6^Office for Open Innovation, Keio University, Tokyo, Japan; ^7^Akasaka Clinic, Tokyo, Japan; ^8^Frontier Business Office, Sumitomo Pharma Co., Ltd., Tokyo, Japan; ^9^Asaka Hospital, Koriyama, Japan; ^10^Oizumi Hospital, Tokyo, Japan; ^11^Department of Psychiatry, Tsurugaoka Garden Hospital, Tokyo, Japan; ^12^Nagatsuta Ikoinomori Clinic, Yokohama, Japan

**Keywords:** machine learning, depression, wearables, personalized medicine, digital health

## Abstract

**Introduction:**

Few biomarkers can be used clinically to diagnose and assess the severity of depression. However, a decrease in activity and sleep efficiency can be observed in depressed patients, and recent technological developments have made it possible to measure these changes. In addition, physiological changes, such as heart rate variability, can be used to distinguish depressed patients from normal persons; these parameters can be used to improve diagnostic accuracy. The proposed research will explore and construct machine learning models capable of detecting depressive episodes and assessing their severity using data collected from wristband-type wearable devices.

**Methods and analysis:**

Patients with depressive symptoms and healthy subjects will wear a wristband-type wearable device for 7 days; data on triaxial acceleration, pulse rate, skin temperature, and ultraviolet light will be collected. On the seventh day of wearing, the severity of depressive episodes will be assessed using Structured Clinical Interview for DSM-5 (SCID-5), Hamilton Depression Rating Scale (HAMD), and other scales. Data for up to five 7-day periods of device wearing will be collected from each subject. Using wearable device data associated with clinical symptoms as supervisory data, we will explore and build a machine learning model capable of identifying the presence or absence of depressive episodes and predicting the HAMD scores for an unknown data set.

**Discussion:**

Our machine learning model could improve the clinical diagnosis and management of depression through the use of a wearable medical device.

**Clinical trial registration:**

[https://jrct.niph.go.jp/latest-detail/jRCT1031210478], identifier [jRCT1031210478].

## Introduction

Currently, few biomarkers can be used clinically to diagnose and assess the severity of mental disorders (1, 2). Blood samples, radiographs, computed tomography (CT), nuclear magnetic resonance imaging (MRI), and numerous other tests are used in psychiatry, but these tests are performed primarily to rule out organic and symptomatic mental disorders. Examinations performed purely for psychiatric diagnostic purposes or as adjuncts to such tests include spinal fluid examinations for meningitis and encephalitis, electroencephalography for the diagnosis of epilepsy, cerebral blood flow scintigraphy and cerebral dopamine transporter scintigraphy for the diagnosis of dementia, and near-infrared spectroscopy (NIRS) for the diagnosis of depression, bipolar disorder, and schizophrenia. However, these examinations represent only a small part of the overall field of psychiatry; in general clinical practice, emphasis continues to be placed on medical interviews. Under these circumstances, when using operational diagnostic criteria such as the Diagnostic and Statistical Manual of Mental Disorders (DSM-5), the diagnosis of a disease is based on the patient’s subjective information, such as medical history, symptoms, and the presence or absence of disturbances in daily life. In addition, the severity of the disease is evaluated based on the patient’s or the evaluator’s own subjectivity using a rating scale. However, such symptom evaluation and diagnoses are not objective, and a large range of variation can exist depending on the judgments of medical professionals, which is a major problem in the field of psychiatry (3, 4).

In recent years, studies using accelerometer-equipped actigraphs have been conducted to quantify sleep disturbances and decreased activity levels that often occur in patients with mental disorders (5, 6). Since the presence of insomnia and decreased activity are known to be risk factors for depression (7, 8) and since it has been suggested that activity and sleep measurements are useful for the assessment and monitoring of mental disorders (9, 10), the quantification of these factors could enable risk detection and severity assessments for depression (11, 12). However, many of the existing studies examining actigraphic monitoring of depressed patients have not collected adequate information on disease severity and treatment status and have not been able to determine the risk of depression or to assess the severity of depression in individual patients (13).

On the other hand, the latest wearable devices are capable of previously unavailable measurement modalities, such as heart rate, respiration rate, skin temperature, and location, in addition to actigraphy functions similar to those used for research, and research on depression is now being conducted using these new devices (14, 15). Our research group previously conducted a study to construct a machine learning model using XGBoost to predict the presence of depression and the severity of depressive symptoms by collecting data from 45 patients with depression and bipolar disorder and 41 healthy subjects who were asked to wear a wearable device (16). The study collected data on seven items (acceleration, pulse rate, skin temperature, ultraviolet light, number of steps, energy expenditure, physical activity, and sleep duration) for 5,250 days. In this manner, a model to predict the presence of depression with an accuracy of 76% and a model to predict Hamilton Depression Rating Scale (HAMD) scores (a rating scale for depressive symptoms) with a correlation coefficient of 0.61 were constructed (16). Although these results were preliminarily based on a limited number of subjects, we believed that this machine learning approach could reach a clinically useful level once more data had been accumulated. In addition, since other previous studies have shown that heart rate variability is useful for identifying mood changes and depression (17, 18), the learning model might be further improved by the addition of this parameter.

In the presently proposed study (SWIFT study: SoftWare development Integrating wearable technologies for Future depression Treatment), which is funded by the Japan Agency for Medical Research and Development (AMED), we plan to construct a machine learning model to detect depressive episodes in subjects with depressive symptoms and to quantify the severities of such episodes. This research aims to develop software capable of being certified as a medical device in Japan by increasing the number of samples and improving the accuracy of the model to be built, compared with models produced in previous studies. The model developed in this study is expected to be widely used in commercially available wearable devices, which could then be used by psychiatrists as a basis for making diagnostic decisions and by non-psychiatric clinicians and occupational physicians as a basis for referrals to psychiatrists.

### Research objectives

The general objective of this study is to build a machine learning model that can be implemented as software in a wearable device to detect depressive episodes in subjects with depressive symptoms and to quantify the severities of such episodes. Our specific objectives are: (1) to obtain triaxial acceleration data, pulse rate, skin temperature, and ultraviolet light data from patients with depressive symptoms and from healthy subjects who have been asked to wear a wristband wearable device; (2) to make diagnoses using the Structured Clinical Interview for DSM-5 (SCID-5) and severity assessments using HAMD and to correlate these diagnoses with the collected data; and (3) to construct an optimal learning model enabling the detection of depressive episodes and the evaluation of depressive episode severity by applying machine learning to the collected data set.

## Methods and analysis

### Trial design and setting

This study will be an open-label observational study. Patients who meet the inclusion criteria will be recruited at the time of their outpatient visit to Keio University Hospital or other medical facilities or during their hospitalization, and healthy individuals without a history of mental illness will be recruited through recruitment advertisements on a website. Patient recruitment will be conducted at the following locations and medical facilities: Tokyo (Keio University, Akasaka Clinic, Oizumi Hospital, Oizumi Mental Clinic, and Tsurugaoka Garden Hospital); Kanagawa (Nagatsuta Ikoinomori Clinic); Fukushima (Asaka Hospital); and Yamagata (Sato Hospital). Other medical facilities may be added depending on the recruiting situation.

In this study, patients with depressive symptoms (depressive disorder, bipolar disorder and related disorders, anxiety disorders, obsessive-compulsive and related disorders, trauma- and stressor-related disorders, dissociative disorders, or somatic symptoms and related disorders) and healthy subjects will wear a wristband wearable device up to five times for 7 days at a time. The timing of the start of the measurement period and the interval between each 7-day cycle will differ for each subject.

### Eligibility criteria

The inclusion criteria for patients shall be as follows: (1) a clinical diagnosis of depressive disorder, bipolar or related disorder, anxiety disorder, obsessive-compulsive or related disorder, trauma-and stressor-related disorder, dissociative disorder, or somatic symptom or related disorder according to the DSM-5 criteria, the presence of depressive symptoms, and an outpatient or inpatient status at Keio University Hospital or another medical facility participating in the study; (2) an age of 18 years or older at the time of consent; (3) confirmation from a psychiatrist that the subject is capable of providing written consent or that a guardian can provide consent if the psychiatrist determines that obtaining consent from the patient will be difficult; and (4) ownership and ability to use a smart phone (iOS 11. 0 or later or Android version 5.0 or later). The inclusion criteria for healthy subjects shall be as follows: (1) no history of psychiatric illness and cooperation with the study on a volunteer basis (after obtaining consent, the M.I.N.I. will be used to confirm that the subject does not have a psychiatric illness); (2) an age of 18 years or older at the time of consent; and (3) ownership and ability to use a smartphone (iOS 11.0 or later or Android version 5.0 or later).

The exclusion criteria for patients shall be as follows: (1) an inability to complete measurements that could affect their medical conditions, as determined by a psychiatrist; (2) the presence of comorbidities that could affect the measurement results, such as paralysis of the upper limbs; and (3) ineligibility for other reasons determined by the principal investigator or sub-investigator. The exclusion criteria for healthy subjects shall be as follows: (1) the presence of comorbidities that could affect the measurement results, such as paralysis of the upper limbs; and (2) ineligibility for other reasons determined by the principal investigator or sub-investigator. Researchers will obtain written informed consent from all the participants. The participants will be able to leave the study at any time.

### Primary outcomes

The primary outcome measures will be the machine-learning algorithm’s estimation of whether the patient meets the DSM-5 definition of a depressive episode and the HAMD machine-learning algorithm’s total score estimate.

### Secondary outcomes

Secondary outcome measures will include the HAMD, Montgomery Asberg Depression Rating Scale (MADRS), Young Mania Rating Scale (YMRS), and Beck Depression Inventory–Second Edition (BDI-II) sub-item scores estimated using machine learning algorithms and the estimated total score of combined scores for multiple items.

### Sample size

**FIGURE 1:**
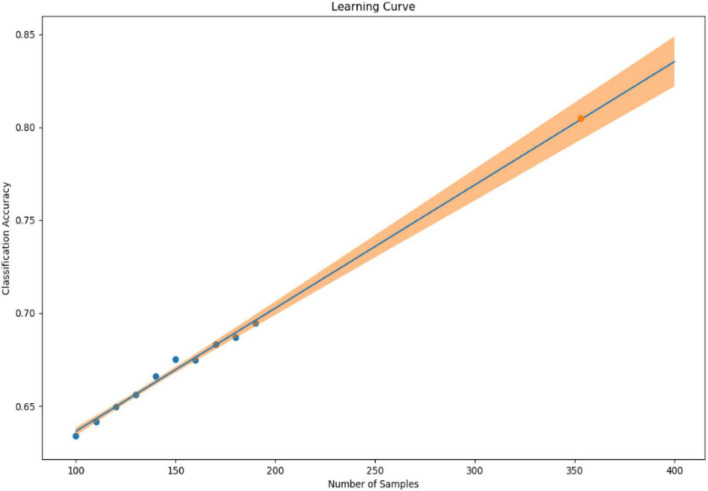
Sample size (X-axis) and classification accuracy (Y-axis).

**FIGURE 2:**
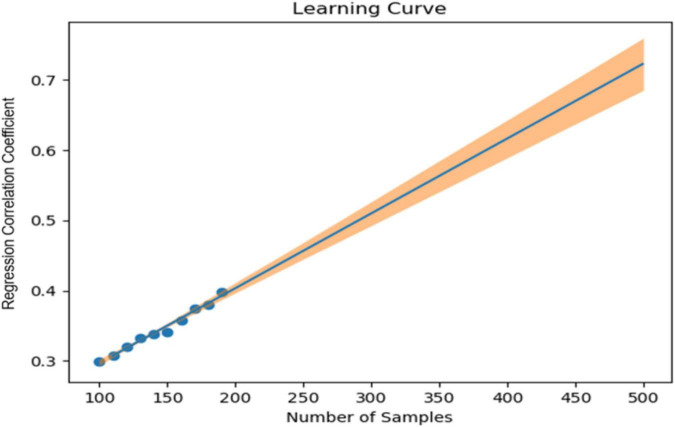
Sample size (X-axis) and correlation coefficient for the 17-item version of the Hamilton Depression Rating Scale (HAMD) measured values and the estimated values (Y-axis).

**FIGURE 3:**
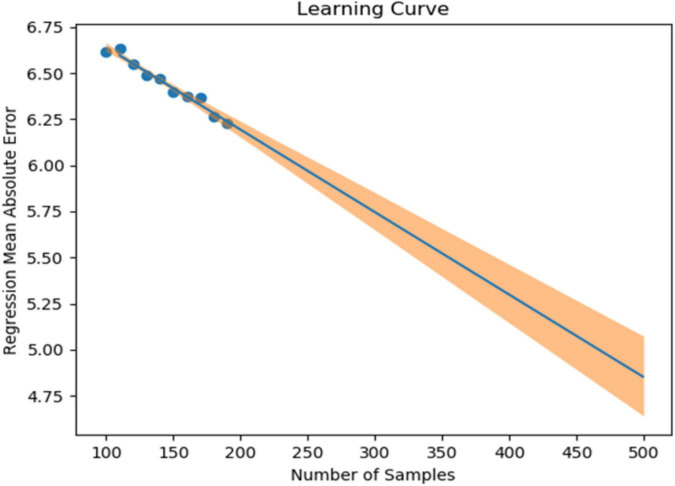
Sample size (X-axis) and mean absolute error for the 17-item version of the Hamilton Depression Rating Scale (HAMD) actual values and estimated values (Y-axis).

In our previous study, we collected 241 datasets over 7 days from 86 patients (45 depressed patients and 41 healthy subjects). In the learning model created using XGBoost, the accuracy of identifying symptomatic depressed patients was 76%, and the correlation coefficient with the 17-item version of HAMD for the severity assessment was 0.61 (16). To improve the accuracy of machine learning, as many datasets as possible should ideally be used. However, to minimize the burden on the research subjects and in consideration of study feasibility, etc., we created a learning curve based on the accuracy of detection models and severity assessment models using various sample sizes from the previous study mentioned above. As a result, about 500 data sets were estimated to be necessary to achieve a rough target of 90% for the detection accuracy of depressive episodes (Figure 1). Additionally, about 650 data sets were estimated to be necessary to achieve a severity assessment accuracy corresponding to a correlation coefficient of about 0.85 between the measured and estimated HAMD values (Figure 2). Furthermore, when the average absolute error from the measured HAMD value was set at three, about 800 data sets were estimated to be necessary (Figure 3). These estimates were based on the results of typical and ideal data collections. For detection purposes, a training data set should ideally contain equal numbers of data sets that meet the criteria for depressive episodes and data sets that do not meet the criteria for depressive; for the severity estimation, the data sets should be evenly distributed across a wide range of severities. In practice, however, symptom severity generally declines during follow-up, even if it was severe at baseline, resulting in a large number of data sets with less severe states. For this reason, the severity of the disease cannot be completely equalized. In the proposed study, we plan to collect data from each research subject for up to five measurement periods, if they are willing to participate. To reach the aforementioned target number of data sets, the numbers of patients with each disorder and healthy controls were determined as follows: 220 patients with depression or bipolar disorder, 40 patients with other psychiatric disorders, and 40 healthy subjects, for a total of 300 patients. However, once the target number of cases or half of the data set has been collected, the accuracy of the detection of depressive episodes and the accuracy of the HAMD-17 estimation will be verified, and the target number of cases will be adjusted upward or downward according to Adaptive Design.

### Data collection methods

The data collection schedule is shown in Table 1. Subjects will be asked to wear a wristband wearable device. On day 7 of each wearing period, (1) the SCID-5 will be used to assess whether the subjects meet the criteria for a depressive episode as defined by the DSM-5 and (2) the HAMD will be used to assess the severity of the depressive episode. These evaluations will be tied to the wearable device data that is being acquired at the same time. A maximum of five evaluations will be conducted per subject, with seven consecutive days regarded as a single evaluation period. The Silmee W22 (TDK Corporation, Tokyo, Japan) wearable device, which is commercially available as a non-medical device, will be used in this study. Body movement and sleep time can be calculated from the Silmee’s accelerator data. Heart rate and heart rate variability can also be measured by processing pulse wave signals collected by Silmee’s photoplethysmographic sensor.

**TABLE 1:** Schedule for data collection and evaluations during the observation period.

Assessment^a^	Visit 0 (Baseline)	Visit 1	Visit 2	Visit 3	Visit 4	Visit 5
Outpatients	Conducted on a regular outpatient basis					
Inpatients	Conducted at least one week apart; after hospital discharge, conducted during outpatient visits					
Healthy subjects	Conducted about once a month (approximately)					
Wearable devices	Wearing the Silmee for at least 7 days prior to the next visit during the study period					
Obtaining consent	◯					
Patient background	◯					
SCID (Patients only)	◯^b^		△	△	△	△
DSM-5 with or without depressive episode (SCID depressive episode part only)		◯	◯	◯	◯	◯
M.I.N.I (Healthy subjects only)	◯	△	△	△	△	△
Clinical Global Impression of illness Severity (CGI-S) (Patients only)		◯	◯	◯	◯	◯
HAMD17		◯	◯	◯	◯	◯
MADRS		◯	◯	◯	◯	◯
YMRS		◯	◯	◯	◯	◯
BDI-II		◯	◯	◯	◯	◯
Concomitant medications	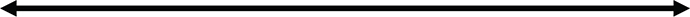					

^a^During the study period, assessment periods will be conducted one to five times per research subject. Therefore, Visits 2, 3, 4, and 5 may not be conducted, depending on the research subjects.

^b^Since it takes time to fully implement the SCID, it will be performed at either visit 0 or visit 1, depending on the convenience and desires of the subject.

△This evaluation will not necessarily be performed every time but will be re-performed as needed (for example, when new symptoms appear or when the diagnosis changes).

“◯” Means that the assessment is to be performed.

The Silmee W22 is capable of calculating the pace of walking or running using acceleration data from a 3-axis accelerometer. The data from the Silmee W22 will be transmitted to cloud storage *via* a dedicated app. The only information that will be uploaded to the cloud will be the device number of the Silmee W22 paired with the dedicated app and the data collected through the Silmee W22. Subjects will be asked to install the dedicated app on their own smartphones and to keep it open or to open it at least once every 3 days.

In this study, electronic data capture (EDC) will be used to collect information such as the date of consent, patient background, SCID diagnosis name, symptom rating scale results, and concomitant medications. We will use “cubeCDMS” from CRScube Inc., for the EDC.

### Data management

The data acquired by Silmee W22 is collected in a form that does not identify individuals. These data will be uploaded to the Silmee Connect cloud managed by TDK Corporation. *Via* the free application “Silmee Connect” from TDK Corporation downloaded to the smartphones of the participating study participants. The data uploaded to TDK Cloud will be transferred to the cloud for this research managed by i2medical LLC, while being linked to the device number of the Silmee W22. These data are then downloaded to i2medical LLC’s secure local environment for analysis.

The database will be constructed using the EDC system (cubeCDMS) with the support of CRScube and the Data Science Division of CMIC Corporation, the outsourcing facility, and the system will be equipped with strict security management, including data backup and access rights management. Clinical data obtained through this research will be managed and stored using the EDC system, and will be passed on to researchers at i2medical LLC and Keio University.

### Statistical methods

All research subjects will be included in the analysis. The data from the Silmee W22 will be denoised and feature-engineered to create a machine learning algorithm that is highly correlated with the presence and severity of depressive episode ratings obtained during researcher-conducted interviews with the subjects. For denoising, we will use techniques such as Continuous Decomposition Analysis (CDA), Discrete Decomposition Analysis (DDA), Gaussian filters, and an adaptive color threshold. These methods are not limited to raw data. If a more suitable method for denoising the raw data becomes available, that method will be used. For feature engineering, we will use autocorrelation, cross-correlation, and other analysis methods to extract temporal structures and patterns for data with time delays. Regarding the feature engineering for heart rate variability, parameters such as standard deviation of normal-to-normal intervals (SDNN), root mean square of successive differences (RMSSD), standard deviation of successive RR interval differences (SDSD), NN50, and Estimated Breath Cycle (EBC) will be used as time-domain features, and low-frequency (LF) and high-frequency (HF) peaks, LF and HF power, normalized LF and HF power, LF/HF ratio, etc., will also be used. In addition, we will search for other features that are useful for improving accuracy and will use the optimal methods available.

For machine learning algorithms, we will use meta-models such as Support Vector Machine (SVM), Support Vector Regression (SVR), Gradient Boosting Classification/Regression, and so on; alternatively, we will use Deep Learning to find the method that gives the highest accuracy. The significance level will be set at 0.05.

In addition, structural brain information and brain functional connectivity from collaborators who participated in another study, “Longitudinal MRI study Identifying the Neural Substrates of Remission/Recovery in Mood Disorders” (Approval Number: 20190239) at Keio University and who also participated in this study will be provided to this study. We will conduct an exploratory analysis using biostatistics and machine learning to examine the relationship between the results of an analysis of structural brain information and brain functional connectivity and the amount of daily life activity and other data obtained from the wearable devices.

### Data monitoring

We will follow the recommended guidelines for clinical research in Japan. According to the guidelines, This study does not involve treatment or other interventions, and the establishment of a data monitoring committee is not required. Monitoring will be conducted by CMIC Corporation, the contractor, to ensure that data are handled appropriately.

### Auditing

The audit is outsourced to the CMIC Corporation and is performed independently of the researchers. The audit will be conducted through monthly management meetings with all contractors and monthly individual meetings to confirm that the work described in the protocols and research contracts is being performed correctly.

### Research ethics approval

Our study has received approval from the institutional review board at Keio University School of Medicine. Approval was granted on November 8, 2021. This study is registered in the Japan Registry of Clinical Trials (jRCT) (jRCT1031210478). Participants’ inclusion will be voluntary. Written consent will be obtained from every participant. Participants will be free to withdraw from the study at any time.

### Consent

Participants will be given both an oral explanation and a written file that documents details of the study. Upon understanding, the participant will be asked to give written consent by signing the consent form. Participants might choose to withdraw from the study at any point in time. Should they choose to do so, their data would be destroyed.

### Confidentiality

The data to be used for the study will be assigned serial numbers in the order of registration for this study, and will be linked and anonymized, and the corresponding table will be kept strictly in a lockable locker in the research room of the Keio University School of Medicine. Thereafter, all collected data and patient background information will be managed under a study number and handled in a manner that separates them from personal information. Paper data will be stored in a lockable locker in a laboratory at Keio University School of Medicine, and the principal investigator responsible for the storage of paper data will lock the lock.

After the study, the electronic data will be stored on CD-R, DVD, or an external hard disk, and stored in a lockable cabinet at Keio University School of Medicine. Anonymization forms will be kept by the personal information manager at Keio University. The storage period will be 5 years after the completion of the research report or 3 years after the final publication of the research results.

### Access to data

As described previously, all personal information and data for the experiment are handled by the personal information manager at Keio University.

### Dissemination policy

The results of this study will be presented at scientific meetings and through the publication of papers. Participants and the public will not be individually notified of the results.

## Discussion

The SWIFT study may lead to the development of medical devices as new diagnostic tools in the field of psychiatry, where biomarkers are scarce. If this technology enables the early diagnosis of depression and can identify signs of recurrence, doctors who do not specialize in psychiatry will be able to refer patients to psychiatrists at an early stage, and occupational physicians will be able to take measures such as reducing workload at an early stage. In addition, the use of this technology by psychiatrists will make it easier to provide measurement-based medical care. As the developed technology will be able to indicate the severity of depression, treatment effects will be easier to detect. Utilizing such technology to clarify symptom changes may lead to improvements in the overall quality of medical care. In addition, patients will be able to monitor their own symptoms. Since previous studies that merely provided information on the status of patients’ symptoms improved the patients’ symptoms (19), the introduction of medical devices that enable such symptom monitoring may be effective for patient treatment.

The application of artificial intelligence (AI) in medicine has been accelerating year by year, and as of 2020, when this study was planned, 11 AI medical devices have been approved by regulatory authorities in Japan (20). However, none of them have targeted psychiatry or have used wearable devices. Similarly, the AI-based medical devices approved by the US food and drug administration (FDA) as of January 2020 did not include those mounted on wearable devices or those that can diagnose depression (21). Thus, the development targeted by the present study is highly novel.

One of the limitations of the learning model to be developed in this study is that it may not be sufficiently accurate to diagnosis or assess the symptoms of severely ill people, since the number of severely ill subjects who are able to participate in this study is expected to be limited because the psychiatric symptoms of severely ill people might prevent them from using wearable devices properly. In addition, elderly people might have difficulty operating wearable devices.

In addition, this study will only recruit Japanese subjects; whether the same diagnostic accuracy can be achieved in other ethnic groups is unclear. In addition, although the information obtained by the Silmee wearable device is similar to that of wearable devices used in other studies (22), the accuracy and content of the information obtained may change in the future, as the performances of wearable devices improve. In addition, the ability to mount the system on other wearable devices would be optimal for use in the future.

Since heart rate variability is also known to be associated with anxiety (23), anxiety symptoms apart from depressive symptoms may also influence the results; since in addition to SCID, HAMD, and MADRS also assess anxiety symptoms, we will attempt to isolate the heart rate variability (HRV) features associated with anxiety.

As for other limitation, the unbalanced sample of the two groups and the relatively small sample size may affect the accuracy of the results of the training model. Therefore, we plan to address this by taking an Adaptive Design approach, validating the accuracy of the prediction during the study and increasing or decreasing the target number of participants accordingly.

Finally, a certain number of users may have concerns, such as privacy, about the wearable device itself or may wish to avoid combining wearables with AI (24, 25). Taking such concerns and/or preferences of patients into consideration will be needed from an ethical perspective before real-world clinical implementation.

## Ethics statement

The studies involving human participants were reviewed and approved by the Keio University School of Medicine Ethics Committee. The patients/participants provided their written informed consent to participate in this study.

## Author contributions

TaK conceived the original study concept. TaK and SK designed and managed the study and wrote the initial draft of the manuscript. All authors contributed to the design of the study and the development and implementation of the protocol, critically reviewed the manuscript, approved the final version of the manuscript, and agreed to be responsible for the accuracy and integrity of the work.

## References

[1] Kapur S Phillips A Insel T. Why has it taken so long for biological psychiatry to develop clinical tests and what to do about it? *Mol Psychiatry.* (2012) 17:1174–9. 10.1038/mp.2012.105 22869033

[2] Beijers L Wardenaar K Bosker F Lamers F van Grootheest G de Boer M Biomarker-based subtyping of depression and anxiety disorders using latent class analysis. a NESDA study. *Psychol Med.* (2019) 49:617–27. 10.1017/S0033291718001307 29860945 PMC6393228

[3] Pies R. How “objective” are psychiatric diagnoses?:(guess again). *Psychiatry.* (2007) 4:18–22. PMC2860522 20428307

[4] Aboraya A Rankin E France C El-Missiry A John C. The reliability of psychiatric diagnosis revisited: the clinician’s guide to improve the reliability of psychiatric diagnosis. *Psychiatry.* (2006) 3:41–50. 21103149 PMC2990547

[5] Burton C McKinstry B Tãtar A Serrano-Blanco A Pagliari C Wolters M. Activity monitoring in patients with depression: a systematic review. *J Affect Disord.* (2013) 145:21–8. 10.1016/j.jad.2012.07.001 22868056

[6] Luik A Zuurbier L Direk N Hofman A Van Someren E Tiemeier H. 24-hour activity rhythm and sleep disturbances in depression and anxiety: a population-based study of middle-aged and older persons. *Depression Anxiety.* (2015) 32:684–92. 10.1002/da.22355 25693731

[7] Riemann D Voderholzer U. Primary insomnia: a risk factor to develop depression? *J Affect Disord.* (2003) 76:255–9. 10.1016/S0165-0327(02)00072-1 12943956

[8] Mammen G Faulkner G. Physical activity and the prevention of depression: a systematic review of prospective studies. *Am J Prev Med.* (2013) 4:649–57. 10.1016/j.amepre.2013.08.001 24139780

[9] Marzano L Bardill A Fields B Herd K Veale D Grey N The application of mHealth to mental health: opportunities and challenges. *Lancet Psychiatry.* (2015) 2:942–8. 10.1016/S2215-0366(15)00268-0 26462228

[10] Reinertsen E Clifford G. A review of physiological and behavioral monitoring with digital sensors for neuropsychiatric illnesses. *Physiol Meas.* (2018) 39:05TR01. 10.1088/1361-6579/aabf64 29671754 PMC5995114

[11] Dogan E Sander C Wagner X Hegerl U Kohls E. Smartphone-based monitoring of objective and subjective data in affective disorders: where are we and where are we going? systematic review. *J Med Internet Res.* (2017) 19:e262. 10.2196/jmir.7006 28739561 PMC5547249

[12] Patel M Foschini L Kurtzman G Zhu J Wang W Rareshide C Using wearable devices and smartphones to track physical activity: initial activation, sustained use, and step counts across sociodemographic characteristics in a national sample. *Ann Intern Med.* (2017) 167:755–7. 10.7326/M17-1495 28973116

[13] Tazawa Y Wada M Mitsukura Y Takamiya A Kitazawa M Yoshimura M Actigraphy for evaluation of mood disorders: a systematic review and meta-analysis. *J Affect Disord.* (2019) 253:257–69. 10.1016/j.jad.2019.04.087 31060012

[14] Rohani D Faurholt-Jepsen M Kessing L Bardram J. Correlations between objective behavioral features collected from mobile and wearable devices and depressive mood symptoms in patients with affective disorders: systematic review. *JMIR Mhealth Uhealth.* (2018) 6:e165. 10.2196/mhealth.9691 30104184 PMC6111148

[15] Wang R Wang W DaSilva A Huckins J Kelley W Heatherton T Tracking depression dynamics in college students using mobile phone and wearable sensing. *Proc ACM Interact Mob Wearable Ubiquitous Technol.* (2018) 2:1–26. 10.1145/3191775 PMC11501090 39449996

[16] Tazawa Y Liang K Yoshimura M Kitazawa M Kaise Y Takamiya A Evaluating depression with multimodal wristband-type wearable device: screening and assessing patient severity utilizing machine-learning. *Heliyon.* (2020) 6:e03274. 10.1016/j.heliyon.2020.e03274 32055728 PMC7005437

[17] Udupa K Sathyaprabha T Thirthalli J Kishore K Lavekar G Raju T Alteration of cardiac autonomic functions in patients with major depression: a study using heart rate variability measures. *J Affect Disord.* (2007) 100:137–41. 17113650 10.1016/j.jad.2006.10.007

[18] Faedda G Ohashi K Hernandez M McGreenery C Grant M Baroni A Actigraph measures discriminate pediatric bipolar disorder from attention-deficit/hyperactivity disorder and typically developing controls. *J Child Psychol Psychiatry.* (2016) 57:706–16. 10.1111/jcpp.12520 26799153 PMC4873411

[19] Snippe E Simons C Hartmann J Menne-Lothmann C Kramer I Booij S Change in daily life behaviors and depression: within-person and between-person associations. *Health Psychol.* (2016) 35:433. 10.1037/hea0000312 26690641

[20] Aisu N Miyake M Takeshita K Akiyama M Kawasaki R Kashiwagi K Regulatory-approved deep learning/machine learning-based medical devices in Japan as of 2020: a systematic review. *PLoS Digital Health.* (2022) 1:e0000001. 10.1371/journal.pdig.0000001 PMC9931274 36812514

[21] Benjamens S Dhunnoo P Meskó B. The state of artificial intelligence-based FDA-approved medical devices and algorithms: an online database. *NPJ Digital Med.* (2020) 3:118. 10.1038/s41746-020-00324-0 32984550 PMC7486909

[22] Lee S Kim H Park MJ Jeon H. Current advances in wearable devices and their sensors in patients with depression. *Front Psychiatry.* (2021) 12:672347. 10.3389/fpsyt.2021.672347 PMC8245757 34220580

[23] Chalmers J Quintana D Abbott M Kemp A. Anxiety disorders are associated with reduced heart rate variability: a meta-analysis. *Front Psychiatry.* (2014) 5:80. 10.3389/fpsyt.2014.00080 25071612 PMC4092363

[24] Segura Anaya L Alsadoon A Costadopoulos N Prasad P. Ethical implications of user perceptions of wearable devices. *Sci Eng Ethics.* (2018) 24:1–28. 28155094 10.1007/s11948-017-9872-8

[25] Tran V Riveros C Ravaud P. Patients’ views of wearable devices and AI in healthcare: findings from the ComPaRe e-cohort. *NPJ Digital Med.* (2019) 2:53. 10.1038/s41746-019-0132-y PMC6572821 31304399

[26] Kishimoto T Kinoshita S Kikuchi T Bun S Kitazawa M Horigome T Development of medical device software for the screening and assessment of depression severity using data collected from a wristband-type wearable device: SWIFT study protocol. *medRxiv.* [Preprint]. (2022). 10.3389/fpsyt.2022.1025517 PMC9811592 36620664

